# Atrophic inferior vena cava is a marker of chronicity of intra-filter and inferior vena cava thrombosis: based on CT findings

**DOI:** 10.1186/s12872-018-0799-z

**Published:** 2018-04-11

**Authors:** Liang Chen, Wanyin Shi, Jianping Gu, Xu He, Wensheng Lou

**Affiliations:** Vascular and Interventional Radiology, Nanjing First Hospital, Nanjing Medical University, No. 68 Changle Road, Nanjing, 210006 China

**Keywords:** Inferior vena cava, Venous thrombosis –Vena cava filter, Computed tomography

## Abstract

**Background:**

A permanently indwelling filter in the inferior vena cava (IVC) may induce caval thrombosis, which could develop and evolve from an acute to a chronic phase. The differential diagnosis of acute and chronic thromboses determines the treatment strategy. The role of computed tomography (CT) in diagnosing acute and chronic intra-filter and IVC thromboses has not been well established. This retrospective study summarizes the CT signs that indicate acute and chronic phases of intra-filter and IVC thromboses.

**Methods:**

This study included eight patients who developed a lower-extremity deep venous thrombosis (DVT) and were treated with intracaval filter placement as an alternative to anticoagulation and thrombolysis. During the follow-up, all patients developed an intra-filter thrombosis in the IVC confirmed by CT and/or CT venography (CTV). Demographic and CT data of all patients during the follow-up period were collected for analysis.

**Results:**

All patients had normal-appearing IVCs prior to filter placement, as shown on trans-femoral venography. Eight filters (five TrapEase, three OptEase) were placed in the eight IVCs, respectively. Subsequently, IVC-CT or CTV revealed acute intra-filter or IVC thrombosis in all eight patients, manifesting as an intracaval filling defect and thickened IVC wall. Filter protrusion and secondary caval atrophy seen on CT indicated a chronically occluded IVC.

**Conclusions:**

IVC thrombosis may result from filter placement. The chronicity of caval thrombotic occlusion is likely to be associated with filter protrusion and secondary IVC atrophy revealed on CT scans.

## Background

Vena cava filters have been increasingly used in patients with lower-extremity deep venous thrombosis (DVT) to avoid pulmonary embolism [1]. Candidates for filter placement include those with contraindications to anticoagulation or thrombolysis [2]. Filter placement, however, carries a 2–30% risk of intra-filter thrombosis, largely depending on the type of filter used [1–4].

Although intra-filter and inferior vena cava (IVC) thrombosis is sometimes clinically silent, it is most likely associated with certain clinical episodes, such as total caval occlusion and/or recurrent DVT, which are largely dependent on the degree of formation of compensatory collateral vessels [5, 6]. Although it has been well known that IVC thrombosis may progress to chronic occlusion [5, 6], this process is rarely detected by either pathology examinations or imaging modalities. It is also unclear whether a filter buried in the thrombus tends to perforate the IVC wall. We report our findings on acute intra-filter and IVC thromboses and chronically thrombotic occlusion revealed by serialized follow-up IVC-CT and CT venography (CTV).

## Methods

The study protocol was approved by our institutional review board. The consent form was waived because of the retrospective nature of the study.

We conducted a retrospective review of medical charts using the hospital’s electronic database of 562 patients who underwent IVC filter placement between January 1, 2007 and December 31, 2014. These patients had been followed by ultrasonography or ascending venography at half-year intervals during the first year and then once a year thereafter. CT was required only if there was thrombus propagation into the IVC or suspected intra-filter thrombosis. The patients’ records were reviewed between March and May 2015. We identified eight cases of CT-confirmed acute intra-filter and IVC thromboses with filter protrusion and chronic occlusion during the follow-up period for whom we conducted detailed chart reviews. These eight patients (among the 562 reviewed) were included in the study simply because they had a complete set of CT scans that revealed the progression of their intra-filter and IVC thromboses from the acute to the chronic stage. The other patients might have had intra-filter or IVC thrombosis but were excluded because of incomplete sets of CT scans.

All eight patients had acute, lower-extremity DVTs, seven of which were left-sided and one that was right-sided. All DVTs involved iliac veins. Three of the eight patients had additional involvement of the proximal femoral vein, and two had additional involvement of the femoral and popliteal veins, respectively. The underlying diseases or risks for these patients developing a DVT included traumatic intracranial bleeding (*n* = 2), liver dysfunction and recurrent upper gastric bleeding in patients with diffuse hepatic metastasis (*n* = 1) or chronic liver cirrhosis (*n* = 3), and malignant lung carcinoma with recurrent hemoptysis (n = 2). Because of the high risk of causing fatal bleeding, none of these patients was given anticoagulation or thrombolytic therapy. All were subjected to IVC filter insertion to avoid pulmonary embolism.

The main initial symptoms of these patients included swelling on the affected leg (*n* = 8) and calf pain (*n* = 6). After filter placement, all patients had intermittent swelling of the affected leg and later developed swelling of the contralateral leg. Three male patients reported moderate pelvic pain with scrotal swelling. Ascites was found in four patients.

Retrievable filters (OptEase; Cordis Corp., Miami Lakes, FL, USA) were placed in three of the eight patients, but attempts to retrieve the filters were abandoned because extensive IVC thrombosis developed. The other five patients were treated with permanent filters (TrapEase; Cordis Corp.). Seven filters were placed in the IVC via the right femoral vein because of a left-sided DVT. Only one filter was placed via the left side because of a right-sided DVT. The technical success of filter placement was 100% (8/8) with no filter tilting or migration observed on venography immediately after filter placement. The clinical characteristics of the patients are shown in Table 1.

**Table 1:** The clinical characteristic of patients presented

Case No.	The underlying disease	Proximal DVT (Yes/No), Side (L/R)	Filter type
1	Chronic liver cirrhosis	Yes, L	OptEase
2	Lung carcinoma	Yes, L	OptEase
3	Traumatic intracranial bleeding	Yes, L	TrapEase
4	Traumatic intracranial bleeding	Yes, L	OptEase
5	Chronic liver cirrhosis	Yes, L	TrapEase
6	Chronic liver cirrhosis	Yes, L	TrapEase
7	Hepatic metastasis	Yes, L	TrapEase
8	Lung carcinoma	Yes, R	TrapEase

*DVT* deep venous thrombosis

A 16-detector CT scanner (Sensation, Siemens, Erlangen, Germany) was used to inspect the IVCs before August 2012. Since then, a 128-slice second-generation dual-source CT device (Somation Definition Flash; Siemens) has been used to obtain CT scans. Because CT studies were obtained for various clinical indications, there was considerable variability in the study type, the use of contrast agent, and the phase of contrast opacification of the vasculature. All eight patients underwent the first CT examination for a definitive diagnosis of intra-filter and IVC thrombosis. During the follow-up period, two young patients with traumatic intracranial bleeding underwent another CT examination. CT scans were performed in some other patients for various reasons, such as to evaluate the therapeutic response of a tumor, for bone pain, or for ascites, as well as to assess the filter and/or the thrombosis. Overall, the eight patients underwent a total of 90 CT examinations. The most common studies included CT of the abdomen or abdomen/pelvis (68/90, 75.6%), IVC-CTV studies (12/90, 13.3%), CT of the lumbar spine (6/90, 6.7%), and CT of the abdominal aorta (4/90, 4.4%).

All data obtained by contrast-enhanced scanning were transferred to workstations, where thin-section images were reconstructed. Post-processing was then performed using multiple planar reconstruction, volume rendering, and maximum intensity projection. Reconstructed three-dimensional IVC imaging could be rotated arbitrarily online for comprehensive inspection.

CT studies were reviewed on a picture archiving and communication system (PACS) workstation (FirsTech, Hefei, China) by a senior physician (W.S.L.) with 20 years of experience in abdominal imaging. Images were viewed in the axial plane, with additional reviews of coronal and sagittal reformations when appropriate. The data on trans-femoral venography were also viewed on PACS. With the availability of serialized imaging, patient-based comparisons were made in terms of the presence or absence of acute intra-filter and IVC thromboses, filter protrusion, and chronic caval occlusion. The presence or absence of filter protrusion was determined for each filter according to the Society of Interventional Radiology Standards of Practice Committee definition of a filter strut or anchor extending > 3 mm outside the wall of the IVC [2]. In addition, four grades were established to indicate the interaction between the filter and the IVC wall, as described by Oh et al. [7].

## Results

None of patients suffered a pulmonary embolism during the follow-up. However, DVT-related symptoms (e.g., swelling, pain) were not relieved, and new onset of contralateral leg swelling was observed, requiring further CT examination for a diagnosis. Six of the eight patients died during a median follow-up of 21.88 ± 14.48 months (range 9–48 months) because of deterioration due to their underlying diseases.

All patients were managed with elastic stockings beginning from the onset of the DVT. Two patients with traumatic intracranial bleeding survived without symptoms of a neurological deficit and have been followed to date to evaluate their IVC filters. Six months after complete recovery from intracranial bleeding, they were prescribed daily oral anticoagulation with warfarin. The warfarin dose was adjusted to maintain the international normalized ratio at 2.0–3.0. Attempts to recanalize the occlusive IVC using endovascular interventions technically failed due to long-segmental occlusion involving the IVC, iliac vein, and femoral vein. Despite such measures to relieve symptoms, these two patients have suffered from intermittent bilateral leg swelling, heaviness, and skin pigmentation, although no ulceration developed.

**Fig. 1:**
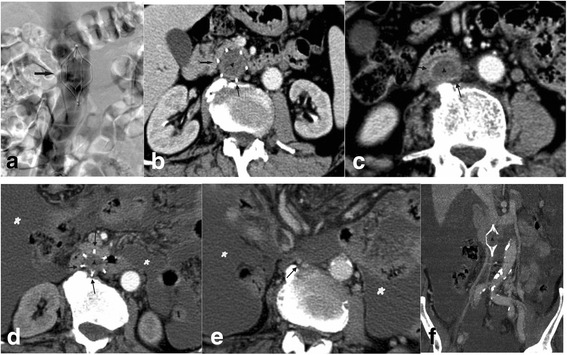
Patient with a typical case of chronic liver cirrhosis who developed a left-sided iliofemoral thrombosis. **a** An OptEase filter (black arrow) was placed in the inferior vena cava (IVC). **b**, **c** Follow-up computed tomography (CT) of the IVC performed 14 days after filter placement revealed a “filling defect” in and below the filter (black arrowhead), indicating extensive intra-filter and IVC thrombosis. The thickened IVC wall (black arrow) appeared as a ring around the thrombus (**c**). **d**–**f** The last CT imaging of the IVC was performed 14 months after filter placement. **d** An intra-filter thrombus (black arrowhead). The protruded struts of the filter (black arrow) indicate grade 2 protrusion. Large amount of ascites (black asterisk) was also present. **e**, **f** The IVC below the filter shows as an “atrophic” strip (black arrow)

**Fig. 2:**
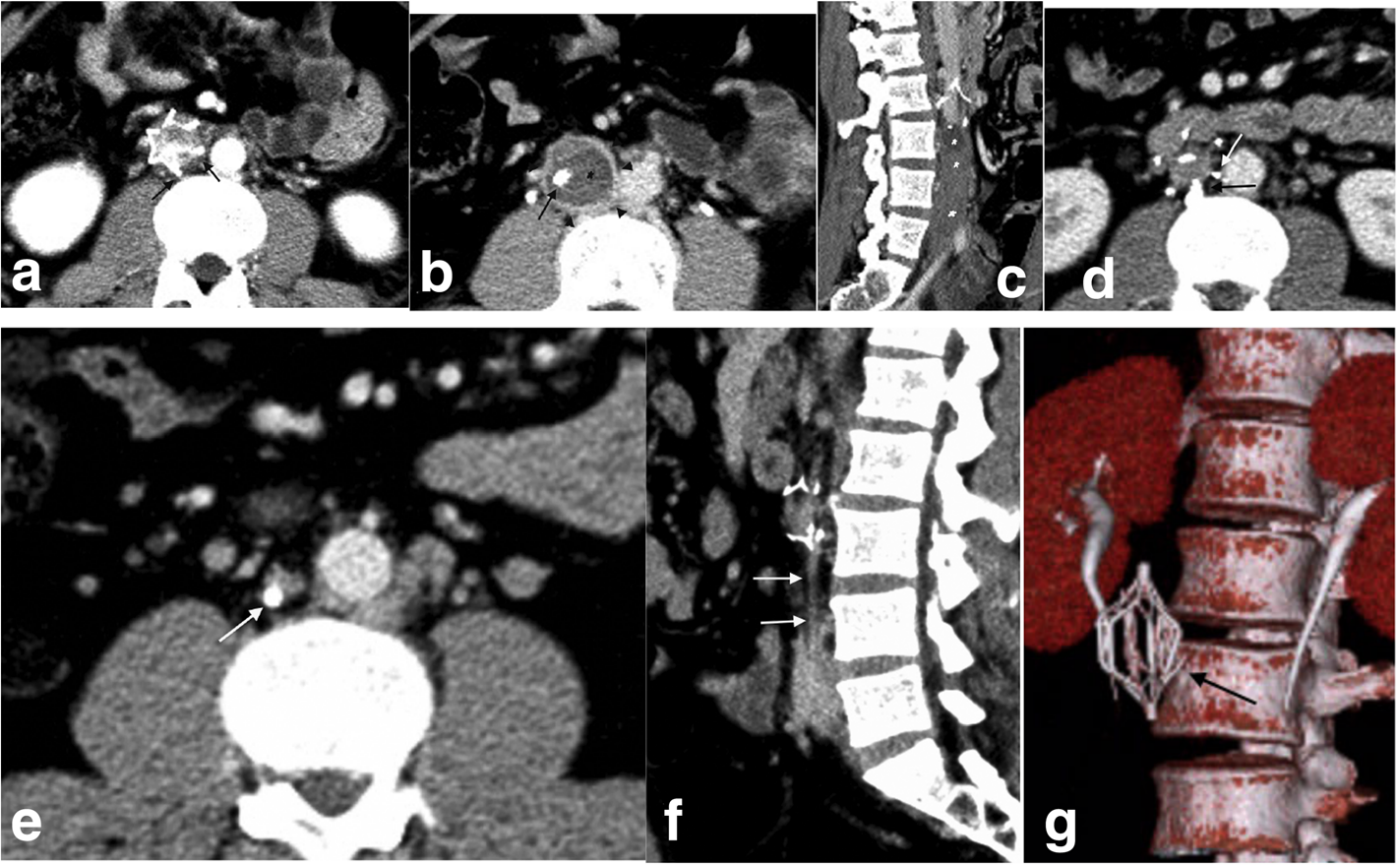
A case of traumatic intracranial bleeding from a left-sided iliofemoral thrombosis. **a**–**c** Follow-up CT of the IVC was obtained 10 days after filter placement. Note the extensive thrombosis (asterisks) in the IVC below the filter and the thickened IVC wall (black arrows) (**b**). The black arrow (**b**) marks the tip of the filter. **d**–**g** The last CT imaging of the IVC was performed outside the IVC shows one of the struts eroding the lumbar body, exhibiting grade 3 protrusion. The IVC has atrophied below the filter (**e**, **f**). Arrow in D marks the tip of the filter. Double white arrows in E mark the IVC as a strip below the filter. The reconstructed CT imaging (**g**) shows a fractured strut of the filter (black arrow)

The median interval between filter insertion and diagnosis of intra-filter and IVC thromboses was 11.62 ± 3.85 days (range 6–18 days). Typical cases are shown in Figs. 1 and 2. The CT features of these patients are shown in Table 2.

**Table 2:** The CT features of patients presented

Case No.	Time to caval thrombosis after filter insertion (days)	Time to filter protrusion first revealed on CT (Months)	Time to chronic caval occlusion first revealed on CT (Months)	Time to the last CT imaging (months)	The Grade of filter protrusion revealed on the last CT imaging ^a^
1	14	3	7	14	2
2	6	5	10	13	3
3	10	8	8	40	3
4	13	2	11	48	2
5	18	4	9	19	2
6	14	3	9	22	2
7	10	8	8	10	2
8	8	3	7	9	3

*CT* computed tomography

^a^The grade of filter protrusion refers to a CT-based classification made by Oh et al. [7]

CT images revealed that the acute thrombosis extended from the iliofemoral vein to the filter basket, represented by a filling defect in the IVC. The IVC was deformed to the point of roundness. The IVC wall was thickened and resembled a ring surrounding the thrombus. The median thickness of the IVC wall was 1.75 ± 0.24 mm (range 1.40–2.10 mm).

During the follow-up, filter protrusion was observed in all eight patients. Five filters (TrapEase) showed protruding struts, and three filters (OptEase) showed a protruding tip outside the IVC wall. One filter (OptEase) showed fractured filter components on CT. During the follow-up period, the severity of filter protrusion progressed from grade 1 to 2 in five patients and from 1 to 3 in three patients. It continued to progress over time. In one patient, a fractured filter strut was noted to be in contact with a vertebral body, producing focal bone erosion that was evident on CT.

Secondary IVC atrophy was observed in all cases, indicating complete occlusion of the IVC. The median interval between filter insertion and apparent IVC atrophy was 8.62 ± 1.41 months (range 7–11 months). The atrophy manifested on CT as a fibroid strip along the original track of the IVC below the filter. The pericaval collateral veins were also visualized on CT. Filter protrusion and secondary IVC atrophy seemed to be associated with the chronicity of the intra-filter and IVC thrombosis.

## Discussion

Eight patients with IVC thrombosis who underwent filter placement were followed with serialized IVC-CT scans or CTV, which revealed an evolving pathway from acute IVC thrombosis to chronic IVC occlusion. During the early stage, the IVC resembled a thickened ring around the thrombus but later took the form of caval atrophy, posing an elevated risk of protrusion of the filter’s components. IVC-CT and CTV proved extremely valuable during the follow-up.

Although this study was retrospective with only a small sample, its findings might help us understand the progression and prognosis of intra-filter and IVC thromboses. Having that information could help us suggest a time point at which to interrupt the progression to chronic occlusion. Such lesions pose the challenge of whether to perform a surgical correction or endovascular recanalization.

Intra-filter and IVC thromboses are most likely the result of extension from an iliofemoral thrombosis or develop from a thrombus captured in the filter’s basket [8]. In the acute setting of IVC thrombosis, the caval wall thickens, indicating extensive edema, a reaction to adhesion with the thrombus. This situation also suggests that there may be an inflammatory component in the caval wall, as found in several animal studies [9]. There is scant evidence of finding an inflammatory caval wall in clinical practice, however, most likely because of inadequate utilization of CT or magnetic resonance imaging (MRI) for longitudinal evaluation of intra-filter and IVC thromboses. Also, the type of inflammation seen in the present study might be sterile, as proved by repeated laboratory tests or the clinical presentation. It should be noted that endovascular infection may develop from placement of an IVC filter [10], usually manifesting as fever of unknown origin or persistent bacteremia. In such cases, fluorodeoxyglucose (FDG) positron emission tomography–computed tomography has been found to be sensitive, providing evidence of increased FDG uptake in the IVC filter and caval wall.

It has been a mystery whether there is a definitive pathological event distinguishing IVC thromboses into acute and chronic stages. In clinical practice, the distinction is mainly based on symptoms, imaging evidence, or both, indicating occlusion of > 3 months duration [11]. On IVC-CT or CTV, a chronically occluded ICV may present as a congenital absence or agenesis [12], which would arouse diagnostic confusion, especially in patients without filter placement. For patients with a definitive history of filter insertion, however, such an appearance on CT scans can be typical, decisive evidence suggesting chronic caval occlusion.

Several factors have been verified as being contributory to such filter-related complications, including the filter type, the patient’s sex, and a history of a malignant tumor [13, 14]. None of the relevant studies, however, have considered an occluded IVC itself a contributor. With our limited experience, we suggest that chronically occluded IVC may be susceptible to filter tilting, embedment, and protrusion, probably due to significant reduction of the cross-sectional area of the IVC caused by occlusion. This view is in line with that of Laborda et al. [15], who suggested that a reduced cross-sectional area of the IVC is associated with a higher risk of filter protrusion. Another cause of an occluded IVC might be increased stiffness or fragility of the caval wall due to inflammation or fibrosis.

A chronically occluded IVC can be reconstructed via angioplasty and stent placement, although it has proved difficult, with a technical failure rate as high as 15% [11, 16]. In our experience, the greatest challenge associated with endovascular reconstruction is that the guidewires or catheters might not be able to access the occluded segment. In such cases, open reconstruction or bypass may be an option. It is intuitive that the technical success of endovascular treatment is sometimes more easily achieved before the IVC becomes chronically occluded. Given the capacity to display different stages with different signs, IVC-CT may help determine the optimal timing for endovascular treatment. However, further studies are needed for confirmation.

All eight of the patients in this study were treated with basket-shaped filters, which are associated with a higher risk of intra-filter thrombosis than are conical filters [17]. For patients who require long-term protection against a pulmonary embolism, a long-term optional filter (e.g., Celect, Option, Denali) might be more appropriate. Moreover, if available, the preferred filter is one that could be retrieved via jugular access in case of a proximal DVT.

There are some limitations of the study, starting with its design as a retrospective study with a small sample. In addition, IVC-CTV imaging has some pitfalls, such as mistaking artifacts for an IVC thrombus, and respiratory movement may induce volumetric changes in the IVC on cross-sectional CT imaging. Another concern involves radiation exposure. Also, the use of contrast media carries risks of some complications as well, such as allergy, impaired renal function, and death. Moreover, the filters used in the study were basket-shaped filters. Usually, there is no penetration with this kind of filter, but with post-thrombotic remodeling the IVC could retract, causing filter protrusion on CT imaging. For this reason, we did not use “penetration” to describe this possibility, but “protrusion.” Finally, given the supersensitivity of MRI and angiography for detecting various tissue components and causing no harm from irradiation, these techniques could be helpful for diagnosing IVC thrombosis and chronic occlusion if the optimal techniques are applied.

## Conclusions

Placement of an IVC filter may cause caval thrombosis. Such an intra-filter or IVC thrombosis could progress from an acute stage, presenting as a thickened caval wall surrounding the thrombus, to a chronic caval occlusion, presenting as an atrophic strip. Chronically occluded IVC, usually coupled with a reduced cross-sectional area, may be a contributory factor for filter-induced protrusion. IVC-CTV well depicts such changes via serialized scanning during the follow-up period.

## Abbreviations

CT: Computed tomography
CTV: Computed tomography venography
DVT: Deep vein thrombosis
IVC: Inferior vena cava
PACS: Picture archiving and communication system
MRI: Magnetic resonance imaging
FDG: Fluorodeoxyglucose
